# Use of computed tomography (CT) for the diagnosis of mechanical gastrointestinal obstruction in canines and felines

**DOI:** 10.1371/journal.pone.0219748

**Published:** 2019-08-23

**Authors:** Brianna M. Miniter, Andréia Gonçalves Arruda, Joshua Zuckerman, Ana V. Caceres, Ron Ben-Amotz

**Affiliations:** 1 Veterinary Specialty and Emergency Center, Small Animal Surgery Department, Levittown, PA, United States of America; 2 College of Veterinary Medicine at The Ohio State University, Department of Veterinary Preventive Medicine, Columbus, OH, United States of America; 3 Cape Cod Veterinary Specialists, Surgery Department, Buzzards Bay, MA, United States of America; 4 Veterinary Specialty and Emergency Center Diagnostic Imaging Department, Levittown, PA, United States of America; 5 Veterinary Specialty and Emergency Center, Small Animal Surgery Department, Philadelphia, PA, United States of America; Harvard Medical School Teaching Hospital, UNITED STATES

## Abstract

The objective of this study was to describe the use of computed tomography (CT) for diagnosis of mechanical gastrointestinal (GIT) obstruction in canines and felines. Medical records of 130 canines and felines that underwent an abdominal CT scan between 2013 and 2015 at a specialty referral hospital for suspected gastrointestinal tract (GIT) obstruction were reviewed. Images were evaluated by a single board-certified radiologist for the presence of foreign material, evidence of obstruction, and location of foreign material present. Confirmation of CT findings was based on surgical exploration or medical management if surgery was not indicated. Of the 97 patients that met the inclusion criteria, 48 (49.48%) had evidence of foreign material present within the GIT and 49 (50.52%) did not. Forty-one patients had evidence of mechanical gastrointestinal obstruction. Thirty-nine of these patients had an obstruction due to foreign material; one had an intussusception with no foreign material, and another had obstruction secondary to mucosal thickening. Forty-five patients underwent exploratory laparotomy, and CT findings were confirmed in all patients. The presence of a GIT obstruction was confirmed intra-operatively in 37 patients and lack of obstruction was confirmed in the remaining eight. Non-surgical medical management was pursued for the remaining patients. Based on follow-up client interviews, clinical signs resolved in all of these patients. In conclusion, computed tomography appears to be useful for the diagnosis of GIT obstruction in canines and felines and is a helpful tool for guiding the recommendation for surgical intervention.

## Introduction

Gastrointestinal (GIT) mechanical obstruction is one of the most commonly encountered surgical emergencies in small animal medicine. Patients with GIT obstruction can present with a variety of non-specific clinical signs including vomiting, anorexia, and electrolyte imbalances [1,2]. If left untreated, GIT obstructions may lead to decreased bowel perfusion and bowel wall necrosis, and increasing risk for septic peritonitis and death [1]. Diagnostic imaging plays an important role in the evaluation of these patients and in guiding appropriate treatment recommendations. The initial diagnostic work-up often includes abdominal radiographs; however, if these images are equivocal, controversy exists regarding the need for more advanced imaging modalities such as ultrasound or computed tomography [3].

Survey abdominal radiographs are widely utilized for investigation of suspected GIT obstruction. Objective measurements comparing small intestinal diameter to lumbar vertebrae on abdominal radiographs has been previously reported as 54%-91.7% sensitive [4–7] and 41.7–66% specific [5,6] for detection of mechanical intestinal obstruction. However, the radiologic abnormalities that accompany mechanical obstruction of the small intestine are usually not specific and can vary with the degree, duration, and location of the obstruction [8]. Finck [7] compared small intestinal radiographic characteristics in canines with and without mechanical intestinal obstruction. This study evaluated multiple small intestinal to vertebral body ratios, and ultimately concluded that the pattern of intestinal dilation and comparison of multilevel small intestinal measurements to the height of the L5 vertebra can be helpful in diagnosing mechanical obstruction. However, even when utilizing this method, the radiographs remained subject to high reader variability, even between board-certified radiologists and radiology residents. While Finck [7] published small intestinal to vertebral ratios indicating a dog is “unlikely to be mechanically obstructed” or “very likely to be obstructed,” there was a large grey area of dogs falling between these thresholds that required additional imaging, such as ultrasound or upper GIT series.

Abdominal ultrasound is also frequently utilized for investigation of potential GIT obstruction. Previous studies have reported a sensitivity of 85%-100% [4,9,10] and specificity of 67%- 94% [10,11] for identification of mechanical gastrointestinal obstruction with abdominal ultrasonography. When a definitive diagnosis of intestinal obstruction cannot be made using abdominal radiographs alone, additional diagnostic imaging, such as contrast radiography, abdominal ultrasound, and/or CT imaging are often necessary [12–14].

Computed tomography imaging utilizes a motorized x-ray source to display a two- to three- dimensional representation of anatomic structures in slices of variable thickness. This eliminates superimposition of intestines on one another and other abdominal organs, making intestinal segments easier to evaluate [5]. In humans, CT is a valuable modality for diagnosing and confirming the location of small bowel obstruction with a sensitivity of 94–100% and an accuracy of 90–95% [3,14]. CT has also been shown to be more accurate than both radiography and ultrasonography in the diagnosis of GIT obstruction in humans [15]. Recently, CT has been proposed as a preliminary screening modality for dogs with acute abdominal signs [16]. One study [17] identified CT as a more accurate screening test compared to ultrasonography and survey radiography for differentiating surgical from non-surgical acute abdominal conditions in dogs.

The objective of this study was to assess the accuracy of abdominal CT scan to aid in the diagnosis of gastrointestinal obstruction. The presence and location of foreign material as well as the presence of GIT obstruction were evaluated.

## Materials and methods

### Study population selection and record assessment

Using a retrospective case series study design, medical records of 130 canines and felines that had abdominal CT scans performed between July 2013 and October 2015 at 404 Veterinary and Emergency Referral Hospital were reviewed. For all selected patients, diagnostic imaging was recommended to aid in the diagnosis of suspected mechanical obstruction based on a combination of history of foreign material ingestion, clinical signs such as vomiting and anorexia, and/or physical exam findings suggestive of gastrointestinal foreign body or mechanical obstruction of the bowel, including abdominal discomfort or nausea.

In all patients, CT scans were performed with the animal under sedation or general anesthesia. Sedatives were administered initially, and if the level of sedation was inadequate for immobilization of the patient during the CT, general anesthesia was induced. Due to the difference in clinical status of each patient, the choice of pharmacological agents and doses administered was at the discretion of the veterinarian or attending anesthesiologist. Sedatives included a combination of an α_2_ agonist (3–5 μg/kg IV Dexdomitor, 0.5 mg/mL dexmedetomidine HCl, Zoetis, Kalamazoo MI) or phenothiazine tranquilizer (0.02–0.1 mg/kg IV acepromazine maleate) and an opioid (0.2–0.3 mg/kg IV Torbugesic, 10 mg/mL butorphanol tartrate, Zoetis Canada Inc., Kirkland QC; or 0.05–0.1 mg/kg IV HYDROmorphone HP 10, 10 mg/mL hydromorphone HCl, Sandoz Canada Inc). For patients imaged under general anesthesia, induction was performed with propofol (PropoFlo, Abbott Animal Health UK) and anesthesia was maintained with isoflurane in 100% oxygen.

### Computed tomography process and evaluation

Patients were positioned in dorsal or sternal recumbency and an abdominal CT was performed using a Toshiba, Aquilion 64 slice CT. Dorsal and lateral scout images were obtained and used for planning and collimation. 1-3mm thick (for felines) and 1-5mm thick unenhanced slices for canines of the entire abdomen were acquired using soft tissue and bone algorithms. The use of intravenous contrast (2.2ml/kg of Omnipaque, 240 mgI/mL iohexol, GE Healthcare, Princeton NJ) was at the discretion of the attending veterinarian and was not used in all patients. If administered, the contrast-enhanced study technique was identical to the unenhanced study. Transverse and dorsal and sagittal reformatted images were obtained. All images were reviewed by a board-certified radiologist for the presence of foreign material, presence of obstruction, and location of foreign material. Criteria evaluated by the radiologist to determine if there was small intestine mechanical obstruction included bowel or gastric distention, presence of intraluminal material (gastric or small intestinal) or the presence of luminal construction adjacent to a dilated intestine, as well as presence of peritoneal mottling adjacent to the abnormal intestine. Intraluminal material was defined as the presence of localized atypical matter within the bowel lumen, often surrounded by fluid and gas, which helped in determining the intraluminal location by delineating the bowel wall. A complete obstruction was characterized by orad dilation of the bowel up to the level of the atypical luminal matter. To identify the general location of the obstruction, transverse and dorsal reformatted images were evaluated. The gastrointestinal tract was divided into stomach, duodenum (portion of intestine extending aborally from the stomach to the proximal jejunum located to the left of the mesenteric root), jejunum (defined as the section of intestine between the aborad portion of the duodenum and ileum), ileum (defined as the distal portion of the small intestine aborad to the ileocolic junction) and colon using the landmarks from Miller’s Anatomy of the dog, third edition. Since a clear anatomic landmark was not appreciated between the jejunum and ileum, the orad boundary of the ileum was approximated. The bowel was carefully followed from the stomach to the colon and from the colon to the stomach several times to authenticate the anatomy.

Following the CT scan, exploratory laparotomy was recommended if the images revealed mechanical gastrointestinal obstruction or if there were other intra-abdominal pathologies present that warranted surgical intervention (e.g. bladder stones, splenic mass, etc.) For patients that surgical intervention was not performed, medical management with a combination of intravenous fluid therapy, anti-emetics, gastro-protectants, pain medications, and/or antibiotics was initiated. Follow-up for all patients was completed through discussion with owners, either in-person or by phone, regarding progression of clinical signs. Timing of follow-up ranged between 1–20 (mean 6.8) days post treatment.

Data obtained from the medical records included age and weight at the time of initial evaluation, breed, sex (male, neutered male, female, female spayed), history and clinical signs, presence of foreign material on CT, presence of GIT obstruction on CT, surgical vs. medical management, operative findings, location of the foreign material/ obstruction and post-treatment follow-up. STATA v.14 software (College Station, TX) was used to calculate basic descriptive statistics.

## Results

**Fig 1:**
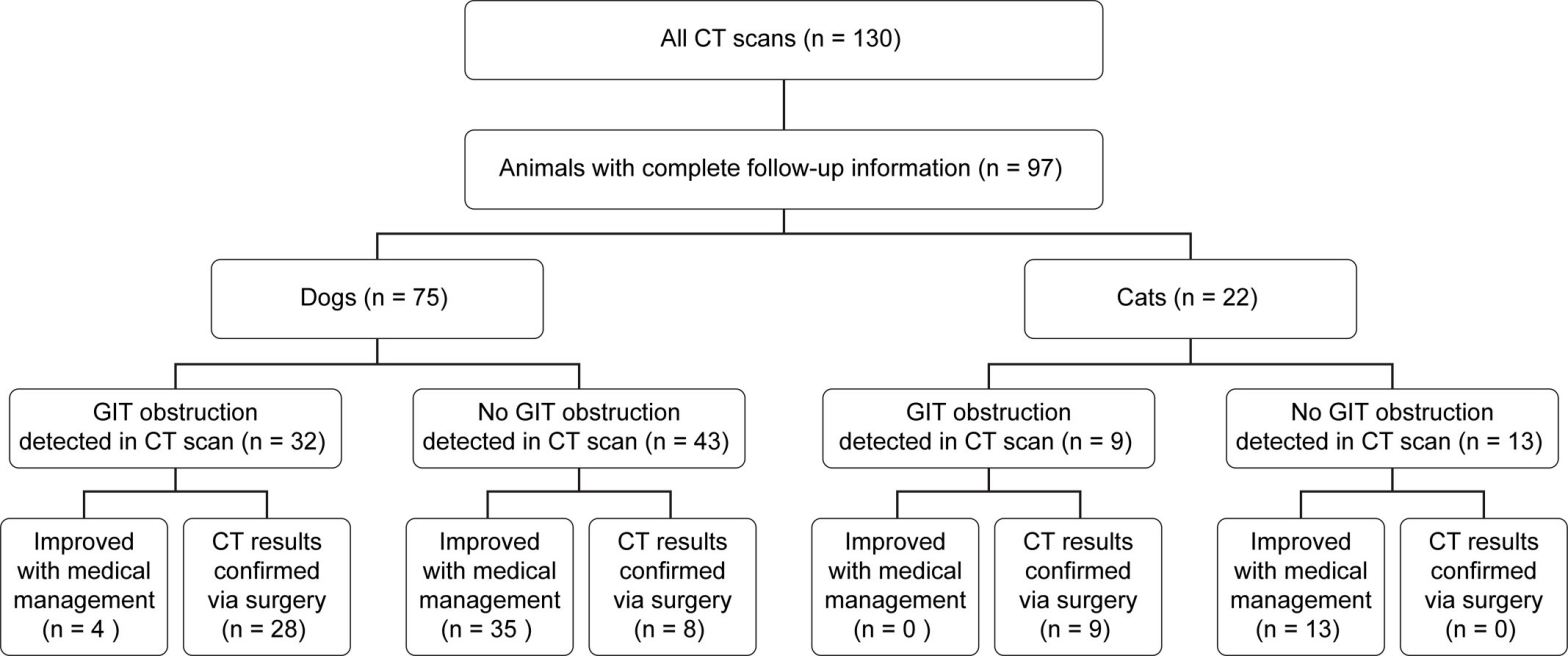
Summary of results by species. Break down of canines and felines that who had GIT obstructions and did not as well as whether or not they improved with medical management or results were confirmed by surgery.

Of the 130 patients that underwent abdominal CT scan, 97 met the inclusion criteria. Thirty-three animals were excluded from the study due to lack of follow-up or death/euthanasia. Of the 33 excluded patients, 23 were euthanized without further diagnostics or treatment, therefore preventing the CT findings from being definitively confirmed or refuted. One patient was excluded due to sudden death without confirmation of underlying condition, and nine patients were excluded from the study due to lack of follow-up. As shown in Fig 1, 97 patients were included in this study; there were 75 canines and 22 felines. There were 3 intact females, 25 spayed females, 13 intact males, and 56 castrated males. Mean age (including canines and felines) was 57.4 months (range 4–168 months) and mean weight was 46.9 lbs (21.3 kg; range 2.2–68 kg). A detailed description of variables of interest by species is provided on Table 1.

**Table 1:** Descriptive statistics for patients included in study.

Variable	Canine (n = 75)	Feline (n = 22)
**Weight (lbs)**		
Mean (standard deviation)	57.55 (30.04)	10.93 (4.42)
Median (inter-quartile range)	62.00 (40.00)	10.00 (8.00)
**Age (months)**		
Mean (standard deviation)	55.95 (43.68)	62.41 (49.67)
Median (inter-quartile range)	36.00 (76.00)	60.00 (60.00)
**Sex (%)**		
Female Intact	4.00 (3/75)	0.00 (0/22)
Female spayed	22.67 (17/75)	36.36 (8/22)
Male intact	17.33 (13/75)	0.00 (0/22)
Male neutered	56.00 (42/75)	63.64 (14/22)
**Findings**		
Foreign body detected on CT	48.00 (36/75)	54.55 (12/22)
Obstruction detected on CT	42.66 (32/75)	40.91 (9/22)
Obstructive foreign body confirmed in surgery^1^	100.00 (28/28)	100.00 (9/9)
Medical management pursued	52.00 (39/75)	59.09 (13/22)
Medical management successful^2^	100.00 (39/39)	100.00 (13/13)

^1^Animals eligible for this outcome were limited to those that had suspected obstruction and underwent exploratory laparotomy

^2^ Animals eligible for this outcome were limited to those that underwent medical management

Following CT interpretation and recommendations of the attending veterinarian, 45/97 (46.39%) patients underwent exploratory laparotomy. In the present study, a total of 41/97 (42.26%) of patients had evidence of GIT obstruction on CT imaging. Surgery was performed in 37/41 (90.24%) patients with suspected GIT obstruction, and surgical findings were in agreement with the CT findings for all operated patients (100.00%). Four of the obstructed patients (10.00%) had suspected pyloric outflow obstruction, and surgical intervention was not elected. For three of these patients, obstructive foreign material within the stomach was consistent with bone fragments. The fourth patient had a pyloric outflow obstruction secondary to gastric mucosal thickening. These four patients were managed medically and clinical signs resolved in all patients. None of the 8/45 (20.00%) patients in which surgery was performed for reasons other than mechanical obstruction had evidence of obstruction noted on the pre-operative CT scan, and this lack of obstruction was confirmed in all patients (100.00%) at the time of surgery.

Forty-eight (49.48%) patients had evidence of foreign material within the GIT (with or without concurrent obstruction), according to the CT scan series. Of these 48 animals, 41 (85.41%) had evidence of gastrointestinal obstruction. Nine out of 97 patients (9.28%) had foreign material within the GIT without evidence of obstruction. In one patient intestinal obstruction was due to an intussusception without foreign material present, in a second patient, the mechanical obstruction was due to thickened gastric mucosa. A detailed description of outcomes of interest by species is provided in Table 1. Furthermore, mechanical GIT obstruction was correctly diagnosed using CT in 100% of patients regardless of patient size. Surgical exploration was not pursued in 52/97 (53.60%) of the patients and medical management was initiated. Based on follow-up, clinical signs resolved in all 52 patients that underwent medical management.

The location of the foreign body within the GI tract was identified by the radiologist in 46 of the 48 (95.83%) patients in which foreign material was present. The suspected location of the GIT obstruction was confirmed via surgery in 35/37 (94.59%) of patients. Abdominal radiographs were performed prior to the CT scans for 70.10% of the patients and 17.53% of patients had abdominal ultrasound prior to the CT scan. Twelve out of the 97 patients (12.37%) animals had both radiographs and abdominal ultrasound prior to CT. Below is a flow diagram summarizing the results.

## Discussion

Limited information exists in the literature regarding the use of CT for the detection of mechanical gastrointestinal obstruction in canines and felines. The findings of the present study suggest that CT may be used to accurately and effectively identify mechanical gastrointestinal obstructions, therefore aiding in the formulation of clinical recommendations (i.e. surgical versus medical management).

Historically, the use of CT in the diagnostic workup for suspected GIT obstruction has been considered cost-prohibitive and typically had to be done under general anesthesia. However, under the conditions of our study, the cost of a CT scan performed under sedation was similar to that of serial abdominal radiographs and overnight hospitalization.

There are limitations to abdominal radiography and ultrasonography for evaluation of GIT mechanical obstruction. While abdominal radiography is often readily available, affordable, and objective measurements comparing small intestinal diameter to L5 have been published, abdominal radiographic changes can also be subjective [7]. Up to 30% of obstructed canines do not have radiographic evidence of obstruction [9]. The images can be inconclusive with identifying the presence of foreign material, whether or not there is GIT obstruction, localization of material/obstruction, and the quality of the radiographic images taken outside our hospital setting were occasionally of mixed or poor quality. All of these factors can influence the decision for recommendation for abdominal exploration.

Limitations of gastrointestinal ultrasonography include experience level of the ultrasonographer, potentially limited availability after hours, and interference of image acquisition or foreign body detection by intraluminal gas. In addition, patient factors, such as body size and amount of intraperitoneal fat, can affect the quality of images acquired [18] and lead to misdiagnosis of obstruction [19]. In a study by Pastore et al. [19], when compared to exploratory laparotomy, abdominal ultrasound failed to identify 35% (5/14) of GIT obstructions [19]. Additionally, 25% of small animals (25/100) had major discrepancies between ultrasonographic and surgical findings [19]. Fields et al. [18] found that CT had an advantage in lesion detection in dogs greater than 25 kg compared to ultrasound, making it a better screening test for abdominal disease [18]. Given these limitations of ultrasonography and radiography, it has been suggested that further diagnostic imaging modalities, such as computed tomography (CT), may be beneficial in diagnosing mechanical gastrointestinal obstruction [11].

A recent article comparing CT and abdominal radiography [5] for the detection of mechanical obstruction in dogs found that CT had a higher sensitivity (95.8% vs. 79.2%) and specificity (80.6% vs. 69.4%) compared to abdominal radiographs reviewed by board certified radiologists; the findings of which were confirmed by exploratory laparotomy. In another report that compared CT to abdominal ultrasonography, CT was a more rapidly attainable and more accurate imaging modality for the diagnosis of mechanical gastrointestinal obstruction in dogs [11]. Although these studies provided important comparative data regarding the differences between CT and other imaging modalities, both utilized relatively small sample sizes and focused exclusively on canine patients.

Computed tomography was equally accurate in the diagnosis of GIT obstruction in both canines and felines, and the accuracy did not appear to be influenced by patient size. The CT findings were confirmed for all patients in which surgery was performed, and for all patients not referred for surgery, medical management was successful. An additional benefit of CT in this study was its efficacy in identifying the presence of foreign material even in patients that were not obstructed–a potential advantage over survey radiography. This was the case in nearly 10% of patients with foreign material present. In these patients, surgery was not recommended, and importantly, all patients demonstrated complete resolution of clinical signs.

Unlike previous studies, our study evaluated a large population of animals [5,11], all records were reviewed by a single board-certified radiologist, therefore minimizing the risk of misclassification bias, and in a large number of patients, it was possible to confirm or refute the CT findings during exploratory laparotomy.

A limitation of this study is variation in follow-up time, particularly as it relates to animals that were treated with medical management. Although all animals that underwent medical management were reported to demonstrate complete resolution of their clinical signs, it was not possible to confirm that these patients did not in fact have a gastrointestinal obstruction that resolved on its own. It is also possible that foreign material causing an intermittent or partial obstruction could have been present; potentially even necessitating surgical intervention at a different hospital after the follow-up had been completed. Furthermore, four patients with images suggestive of obstruction did not undergo surgery; therefore these obstructions could not be confirmed. However, these patients did improve with medical management, suggesting that surgical intervention may not have been indicated.

Finally, patient selection for this study was based on the prerequisite of having a CT scan to evaluate for gastrointestinal obstruction. This specific focus narrowed our eligible patient population; which contributed to the lack of variability in our findings. Furthermore, due to the direct relationship between CT findings and further patient management (e.g. indicated laparotomy), the ability of our study to calculate diagnostic characteristics such as sensitivity and specificity was limited, since what would be our gold standard practice for calculation of such test characteristics (laparotomy) was directly associated with CT findings.

## Conclusions

Computed tomography is a promising diagnostic imaging modality for the evaluation of suspected mechanical GIT obstruction and the presence of foreign material within the gastrointestinal tract, even in patients in which foreign material is present but non-obstructive. Use of CT may aid in the rapid diagnosis of gastrointestinal obstruction and in guiding clinical recommendations regarding the need for surgical intervention.

## Supporting information

S1 File CT FB final (version 1). (XLSX)
